# Efficacy of Single- and Dual-Docking Robotic Surgery of Paraaortic and Pelvic Lymphadenectomy in High-Risk Endometrial Cancer

**DOI:** 10.3390/jpm14050441

**Published:** 2024-04-23

**Authors:** Magdalena Bizoń, Maciej Olszewski, Agnieszka Grabowska, Joanna Siudek, Krzysztof Mawlichanów, Radovan Pilka

**Affiliations:** 1 LUX MED Oncology Hospital, św. Wincentego 103, 03-291 Warsaw, Poland; molszewski.onkolog@gmail.com; 2 Faculty of Medicine, Lazarski University, 02-662 Warsaw, Poland; 3 Neo Hospital, Kostrzewskiego 47, 30-437 Cracow, Poland; 4 Faculty of Mechanical Engineering, Cracow University of Technology, Al. Jana Pawła II 37, 31-864 Cracow, Poland; 5 Faculty of Medicine and Health Sciences, Andrzej Frycz Modrzewski Krakow University, 30-705 Cracow, Poland; 6 Department of Obstetrics and Gynecology, University Hospital, Palacky University Olomouc, 779 00 Olomouc, Czech Republic

**Keywords:** robotic surgery, endometrial cancer, paraaortic lymphadenectomy, dual docking, single docking

## Abstract

(1) The surgical method of choice for the treatment of endometrial cancer is minimally invasive surgery. In cases of high-risk endometrial cancer, completed paraaortic and pelvic lymphadenectomy are indicated. The aim of this study was to analyze the types of docking during robotic surgery assisted with the da Vinci X system while performing paraaortic and pelvic lymphadenectomy. (2) Methods: A total of 25 patients with high-risk endometrial cancer, with a mean age of 60.07 ± 10.67 (range 34.69–83.23) years, and with a mean body mass index (BMI) of 28.4 ± 5.62 (range 18–41.5) kg/m^2^, were included in this study. The analyzed population was divided into groups that underwent single or dual docking during surgery. (3) Results: No statistical significance was observed between single and dual docking during paraaortic and pelvic lymphadenectomy and between the type of docking and the duration of the operation. However, there was a statistically significant correlation between the duration of the operation and previous surgery (*p* < 0.005). The number of removed lymph nodes was statistically associated with BMI (*p* < 0.005): 15.87 ± 6.83 and 24.5 ± 8.7 for paraaortic and pelvic lymph nodes, respectively, in cases of single docking, and 18.05 ± 7.92 and 24.88 ± 11.75 for paraaortic and pelvic lymph nodes, respectively, in cases of dual docking. (4) Conclusions: The robot-assisted approach is a good surgical method for lymphadenectomy for obese patients, and, despite the type of docking, there are no differences in the quality of surgery.

## 1. Introduction

One of the most common gynecological neoplastic diseases in Europe is endometrial cancer. A simple hysterectomy with bilateral salpingo-oophorectomy and bilateral sentinel lymph node detection are recommended according to the ESGO/ESTRO/ESP guidelines. However, complete pelvic and paraaortic lymphadenectomy is indicated in cases of high-risk endometrial cancer. In cases of advanced disease, every suspicious lymph node detected in pre-diagnostic images should be removed. There is no evidence of the therapeutic impact of the full systematic paraaortic and pelvic lymphadenectomy of nonsuspicious lymph nodes [1]. The surgical removal of lymph nodes was first reported in the 1960s for the treatment of endometrial cancer [2,3]. The importance of lymphadenectomy in endometrial cancer was first described by Creasman et al., who reported a correlation between tumor grade, the depth of myometrial invasion, and nodal metastasis. About 10 years after that, the significance of paraaortic lymphadenectomy was mentioned [4]. As a result of the detection of metastasis in lymph nodes, the risk of recurrence can be estimated and an adequate choice for adjuvant treatment can be offered [5,6]. Mariani et al. found that the involvement of pelvic lymph nodes is an independent predictor of prognosis, mortality, and recurrence [7]. Additionally, the involvement of pelvic and paraaortic lymph nodes is a major risk factor for poor prognosis in endometrial cancer [8]. Locoregional treatment is beneficial in these cases and is important for prolonging overall survival and progression-free survival [9,10].

On the other hand, two prospective randomized trials have been published, with no evidence for the correlation of systematic lymphadenectomy or the improvement of progression-free or overall survival in endometrial cancer [11,12]. Lymph node metastasis is an indicator for poor prognosis in cancers.

Minimally invasive surgery is the method of choice according to the ESGO guidelines [1]. Nowadays, procedures assisted with robotic surgery are increasingly common. In 2005, the da Vinci surgical system received approval from the US Federal Drug Administration for the treatment of gynecological cancer [13]. Since 2018, robotic surgery has been available in Poland in some centers. The da Vinci robotic system developed by Intuitive has been produced in several generations. Nowadays, only generations X and Xi are used worldwide.

Upper abdominal surgery for omentectomy and paraaortic lymphadenectomy may require different robot positioning, e.g., one-site and dual docking, depending on the generation of the robotic system.

The aim of this study was to analyze the types of docking used during robotic surgery assisted with the da Vinci X system while performing paraaortic and pelvic lymphadenectomy.

## 2. Materials and Methods

### 2.1. Characteristics of Population

This study was conducted from December 2020 to December 2022. This study included 25 patients with high-risk endometrial cancer after paraaortic and pelvic lymphadenectomy, assisted by the da Vinci X system, was completed. The mean age of the analyzed population was 60.07 ± 10.67 (range 34.69–83.23) years. All patients underwent a hysterectomy and bilateral salpingo-ovariectomy with completed pelvic lymphadenectomy and paraaortic lymphadenectomy. An omentectomy was performed in cases of serous or clear-cell carcinoma. Preoperative investigations included a computed tomography (CT) scan of the thorax and abdomen and magnetic resonance imaging (MRI) of the pelvis.

Inclusion criteria: age above 18 years, high-risk endometrial cancer with pathologically proven cancer of any histological type, clinical stage I-II for surgical treatment, and consent to participate in this study.

Exclusion criteria: age below 18 years, pregnancy, surgical contraindications for the surgical treatment of endometrial cancer, anesthesiologic contraindications to surgical treatment, locally advanced disease or intraabdominal metastases or distant metastases in preoperative diagnostic methods (CT scan and MRI), a bleeding disorder, mandatory antithrombotic treatment, and a lack of consent to participate in the study. Patients who received neoadjuvant treatment (either hormonotherapy or chemotherapy), with primary synchronous cancers, or with incomplete medical records were also excluded from this study.

### 2.2. Docking and Port Placement

**Figure 1:**
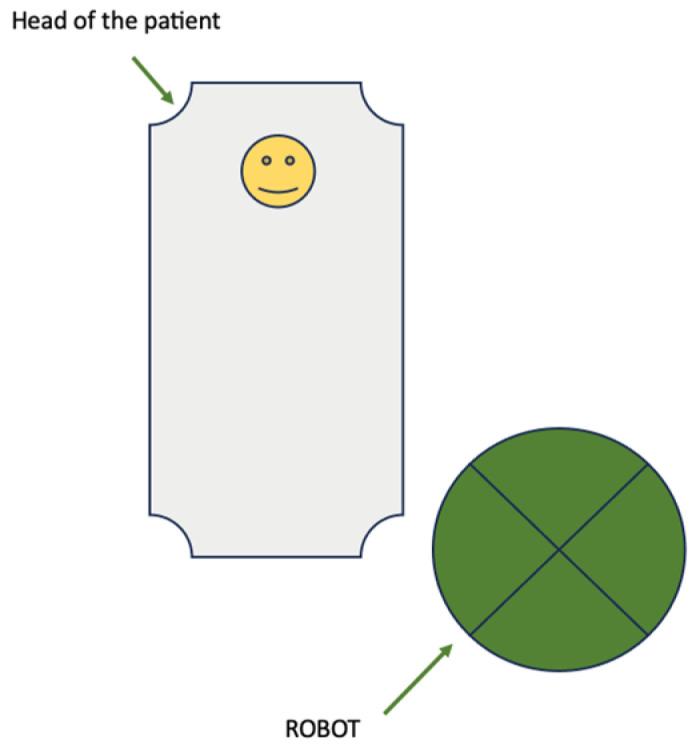
Single-site docking.

Throughout this study, the da Vinci System X was used. Initially, the patient was in the dorsolithotomy position. After the induction of anesthesia, the first trocar was placed below the umbilicus for the camera. Then, the patient was placed in the Trendelenburg position at 25–30 degrees. The rest of the robotic trocars (3) with 8 mm diameter and one assistant trocar with 12 mm diameter were introduced. All trocars were inserted in the same line with 8 cm distance between them. The location of the robot depended on the type of docking, which was randomized for each patient. In the case of single docking, the robot was situated on the left side of the patient [Figure 1]. Instruments were introduced in robotic arms, and the procedures of paraaortic lymphadenectomy and radical hysterectomy could then be performed.

**Figure 2:**
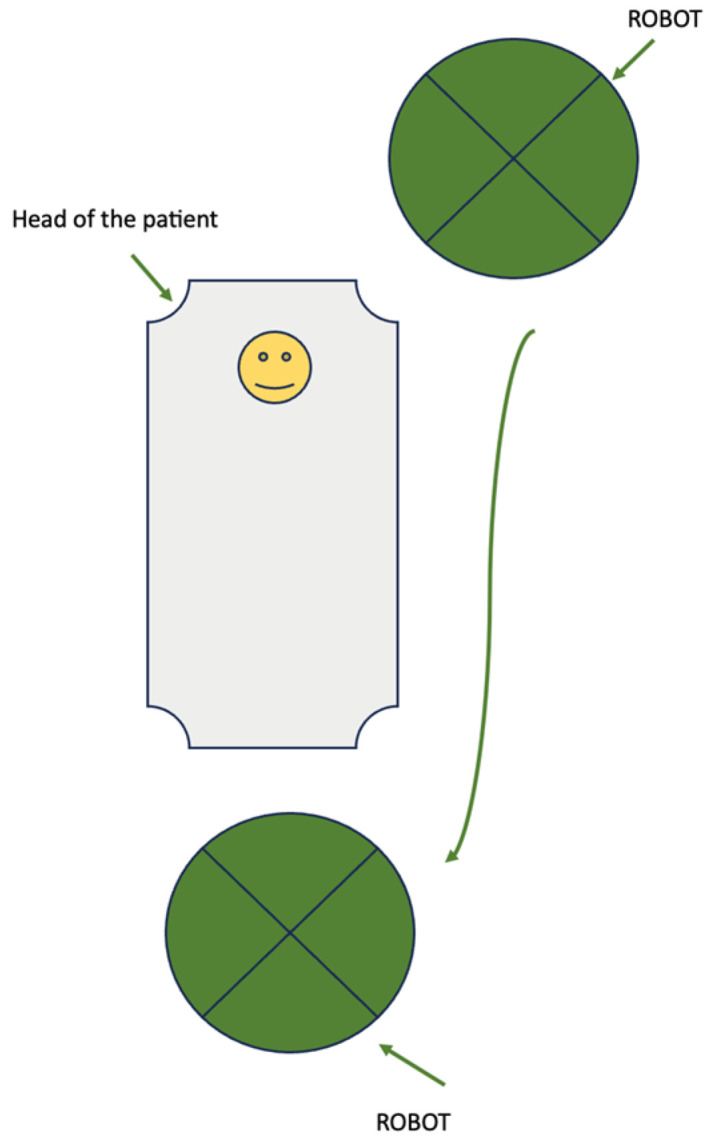
Dual docking.

In dual docking, the robot has two localizations during the operation. Firstly, the robot is located above the head of the patient for paraaortic lymphadenectomy. Once the aortic lymphadenectomy is completed, the robotic system is undocked. Then, the robot is located between the legs of the patient for radical hysterectomy and pelvic lymphadenectomy [Figure 2]. 

If necessary, omentectomy is performed either with one-site or dual docking.

### 2.3. Procedure of Paraaortic Lymphadenectomy

**Figure 3:**
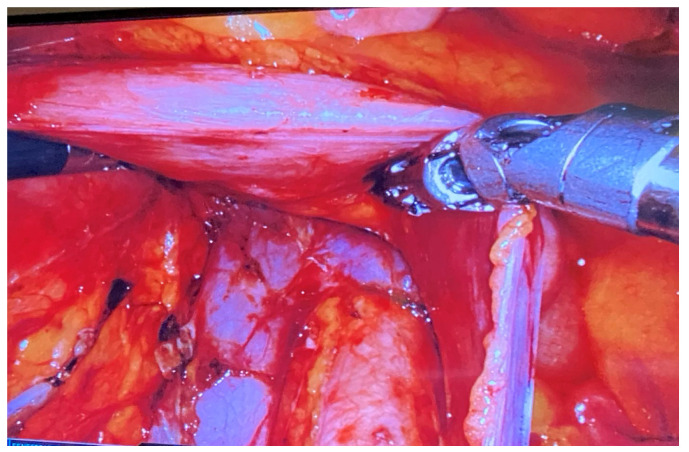
Aortic vessels after lymphadenectomy.

Steps of paraaortic lymphadenectomy in mini-invasive surgery were described by Heinemann [14]. In our procedures, after docking, we opened the peritoneum and performed transperitoneal paraaortic lymphadenectomy. The first step is to identify the right common iliac vessels, right ureter, aorta, vena cava, inferior mesenteric artery, right ovarian vein, left renal vein, and left ureter. All lymph nodes removed from this area were put in an endobag and extracted through the assistant trocar. The next step after paraaortic lymphadenectomy is completed is pelvic lymphadenectomy. Figure 3 presents aortic vessels after lymphadenectomy.

### 2.4. Procedure of Pelvic Lymphadenectomy

Pelvic lymphadenectomy is limited superiorly by the bifurcation of the common iliac vessels, laterally by the psoas muscle, medially by the ureter, inferiorly by the circumflex ilia vein, and posteriorly by the obturator nerve.

All lymph nodes removed from these are put in an endobag and evacuated through the assistant trocar. Histological examination is performed for every removed tissue.

### 2.5. Statistical Methods

MS Excel was used for descriptive analysis based on the mean, median, and standard deviation. Analysis included statistical tests: the non-parametric Mann–Whitney U test, the chi-square test, and the Shapiro–Wilk test. Statistical significance was considered as a *p*-value < 0.05. For statistical analysis, R programming language version 4.1.2 (R Core Team, Vienna, Austria) was used.

## 3. Results

### 3.1. Operation Time and Blood Loss

In the whole analyzed population, eight patients had previous laparotomy. In these cases the mean operation time was 225 (range 170–295) min. The mean operation time in all patients who participated in the study was 196 (range 110–295) min. Longer operation time after previous surgeries was statistically significant (*p* < 0.05). There was no statistically difference in history of surgery and type of docking.

For the rest of the group with no laparotomy in the past the mean operation time was 182 (range 110–250) min.

The mean operation time in single docking was 173 (range 110–235) min, while for dual docking it was 204 (range 130–295) min.

Mean blood loss for the whole analyzed population was 50 (range 5–150) mL. In the case of single docking, it was 50 (range 20–100) mL, and for dual docking, it was 50 (range 5–150) mL. No patient required a blood transfusion. No perioperative complications were registered. The anesthetic management was not modified by the robotic route. No conversion to laparotomy was registered.

There was no difference in blood loss regarding the type of docking. There was no statistically significant effect of age, BMI, or blood loss (*p* > 0.05).

No statistically significant association between previous surgery and type of docking was found, but there was a statistically significant correlation of previous laparotomies and operation time (*p* = 0.0247).

### 3.2. Value of BMI and Lymphadenectomy

For better assessment of the number of lymph nodes regarding BMI, the population was divided into three groups. The first one consisted of five patients with a BMI under 25 kg/m^2^. The mean number of paraaortic lymph nodes was 9.8 (range 7–19), and that of pelvic lymph nodes 13.8 (range 12–16). There were 13 patients with a BMI between 25 kg/m^2^ and 30 kg/m^2^. The mean number of paraaortic lymph nodes was 17.1 (range 6–29), and that of pelvic lymph nodes was 24.5 (range 11–50). The latter group with a BMI above 30 kg/m^2^ was represented by seven women, with a mean number of paraaortic lymph nodes of 21.8 (range 14–35) and a mean number of pelvic lymph nodes of 33 (range 20–46).

There was a statistically significant association between BMI and number of paraaortic lymph nodes (*p* = 0.0152). There was also a statistically significant correlation between BMI and number of pelvic lymph nodes (*p* = 0.0852).

Detailed characteristics of the population regarding BMI are presented in Table 1.

On the other hand, there was no statistically significant association between BMI and the experience of the surgeons (*p* > 0.05). In our study, the surgeons were two gynecologic oncologists with 2 and 12 years of experience in the robot-assisted approach.

No statistically significant correlation of number of lymph nodes and age was found (*p* > 0.05).

### 3.3. Docking and Lymphadenectomy

Dual docking was performed in 17 cases with a mean age of 61.3 (42.08–73.68) and a mean BMI of 28.59 (19.15–41.52) kg/m^2^. Drainage was applied in 14 cases in this group. Mean blood loss was 49.7 (5–150) mL. In this group, seven patients had undergone previous laparotomies. The mean number of paraaortic lymph nodes was 18.05 (7–35) and the mean number of pelvic lymph nodes was 24.88 (11–50). The mean operation time was 204 (130–295) minutes.

Histologically, there were finally diagnosed 14 cases of endometrioid cancer, 1 case of serous cancer, 1 case of clear-cell carcinoma, and 1 case of a mixed histological type (endometrioid + clear-cell carcinoma).

According to the FIGO classification, eight patients were assessed as stage IA, five as stage IB, three as stage II, and one as stage IIIB.

On the other hand, one-site docking was performed in eight cases with a mean age of 57.48 (34.69–83.24) years and a mean BMI of 28.03 (17.97–37.83) kg/m^2^. Drainage was applied in two cases. The mean blood loss was 50 (20–100) mL. In this group, only one patient had undergone laparotomy in the past. The mean number of paraaortic lymph nodes was 15.87 (6–24) and the mean number of pelvic lymph nodes was 24.5 (14–43). The mean operative time was 177 (110–235) min. Histologically, seven patients were finally diagnosed with endometrioid cancer and one was diagnosed with clear-cell carcinoma. 

There was no statistically significant association between single or dual docking and the number of removed lymph nodes, either paraaortic or pelvic. 

### 3.4. Follow-Up

Postoperative staging registered 12 patients with stage IA, 7 with stage IB, 4 with stage II, and 2 patients with IIIB endometrial cancer according to the FIGO classification of endometrial cancer. The characteristics of the population, according to the FIGO classification, are presented in Table 2. 

Follow-up visits were held every 3 months after the completion of adjuvant treatment. In the case of no adjuvant curative effect, observation was continued every 3 months. No recurrence or death was reported throughout the study.

Chemotherapy was introduced as an adjuvant treatment in four cases. Radiotherapy was indicated for two patients. Five patients underwent brachytherapy.

## 4. Discussion

The importance of lymphadenectomy in endometrial cancer has been analyzed in two randomized trials in Europe (the ASTEC trial and an Italian study), which compared pelvic lymphadenectomy and no lymphadenectomy. Both showed no benefits to survival after performing lymphadenectomy [11,12].

On the other hand, lymphadenectomy is strongly recommended in high-risk endometrial cancer. Venigalla et al. observed an improvement in overall survival after pelvic lymphadenectomy or with no pelvic lymphadenectomy in high-risk cancers [15]. Also, Papathemelis et al. in the study in 2017 reported a significant effect of systematic pelvic and paraaortic lymphadenectomy in reduction of recurrence rate and longer overall survival, while in 2018 Papathemelis et al. found lower effect of lymphadenectomy in patients with low grade endometrial cancer [16,17]. 

Konno et al. reported studies with no lymphadenectomy in endometrial cancer, which could be preferred in practice. The SEPAL study showed paraaortic and pelvic lymphadenectomy in high-grade endometrial cancer with no significant difference in complications compared to no lymphadenectomy [18,19]. The SEPAL trial revealed longer survival for patients at intermediate or high risk of recurrence after paraaortic lymph node removal. On the other hand, pelvic lymphadenectomy alone may not be a sufficient procedure for patients with endometrial cancer at risk of lymph node metastasis [20]. In our study, high-grade endometrial cancer was treated by hysterectomies with completed paraaortic and pelvic lymphadenectomy with a robot-assisted approach using da Vinci System X. 

Marek et al. performed a study of paraaortic lymphadenectomy using da Vinci System S and found a higher number of paraaortic lymph nodes with the upper limit at the left renal vein using a dual-docking approach [21]. The same borders of paraaortic lymphadenectomy were introduced in our study. We did not observe complications accompanying completed lymphadenectomy.

The safety and advantages of robotic surgery in paraaortic lymphadenectomy showed Gallotta et al. in the study based on 71 patients with endometrial cancer. The operative time was 210 min; meanwhile, in our study, it was less in both groups. The mean number of paraaortic lymph nodes was 12; meanwhile, in our study, the mean number of paraaortic lymph nodes was higher in single- and dual-docking cases [22].

Lee et al. compared robotic surgery and the laparoscopic approach in paraaortic lymphadenectomy. The operation time was shorter in the robotic approach and the number of infrarenal paraaortic lymph nodes was significantly higher in procedures performed using robotic compared to laparoscopic surgery [13]. In view of that, we decided to compare different types of docking in surgery assisted by a robot and use of the laparoscopic approach in our study.

In 2015, Persson et al. published a study of robotic infrarenal paraaortic and pelvic nodal staging in endometrial cancer. The procedures were performed using the same docking. In 10 cases of 150 patients, conversion to laparotomy occurred because of disseminated disease. They performed paraaortic lymphadenectomy to the left renal vein in 98 cases, to the inferior mesenteric artery in 30 patients, and in 12 aborted removals of paraaortic lymph nodes because of technical or surgical reasons [23]. The mean time of surgery was 200 min; meanwhile, in our study, the mean operation time was 196 minutes. Mean intraoperative blood loss was 50 mL (0–5000), which is the same as that in our research. Sixteen patients developed grade 2 lymphedema of a lower extremity, with half of them being bilateral, while there were no cases of chylous ascites. In our study, we did not observe complications after lymphadenectomy.

In comparison to the mean BMI of 27.3 (16.4–53.3) kg/m^2^, reported by Persson et al., in our study, it was 28.03 kg/m^2^ [24].

Soliman et al. revealed that half of gynecologic oncologists perform paraaortic lymphadenectomy to the limit of the inferior mesenteric artery (IMA) and 11% of them perform it to the renal vessels [25]. In our study, the limit of the paraaortic lymphadenectomy in dual docking was a renal vein; meanwhile, in single-site docking, this limit is technically more difficult. Systematic paraaortic lymphadenectomy seems to be more important while performing above the IMA with the dissection of renal vessels in high-risk patients [7,26].

Ekdahl et al. also described dual docking in robotic surgery of paraaortic lymphadenectomy (PALND) in endometrial cancer. The median total number of lymph nodes was 36 (pelvic—20; paraaortic—16). Operating time was 228 (181–371) min. In seven cases, PALND was aborted because of a technical inability to reach the left renal vein (10%) [27]. In comparison to our study, the mean number of total removed lymph nodes was 43 (pelvic—25; paraaortic—18) with a mean operation time of 204 (range 130–295) min.

Maenpaa et al. performed 283 robot-assisted paraaortic lymphadenectomies using single docking, which were published in 2018. There were seven conversions to laparotomy (2.5%). They assessed the learning curve as more than 40 operations. Paraaortic lymph node involvement was described in 43 cases (15.2%). The total number of paraaortic lymph nodes removed was 12 (range 0–38); meanwhile, in our study, it was 15.8 (range 6–24) in 25 patients with no metastases in the final histological examination. They placed patients in a 28-degree Trendelenburg position and used a zero-degree scope. They also performed omentectomy in 88 cases. The cranial-to-IMA level was achieved in 83% (235 operations) of cases, while the level of the IMA was reached in 7% of cases (19 operations). Lymphadenectomy was carried out below the IMA in only 3% of cases. The median ranges for pelvic and paraaortic lymph nodes were 23 (0–88) and 12 (0–38). Throughout our study, we used a 30-degree camera scope and placed the patient in a 30-degree Trendelenburg position. During procedures performed with one-site docking, the numbers of removed pelvic and paraaortic lymph nodes were 24.5 (range 14–43) and 15.8 (range 6–24), respectively.

In 14 cases, the uterus was removed by mini laparotomy because of a narrow vagina or a large uterus. BMI of 35 kg/m^2^ was the limit of successful lymphadenectomy; in cases with a BMI higher than 35, the complication rate was increased [28]. In our investigation, a few cases with BMI above 35 kg/m^2^ were included. 

Mariani et al. reported that only 2% of patients with negative pelvic lymph nodes had positive paraaortic ones. In our study, we did not detect metastasis in paraaortic lymph nodes [29,30]. On the other hand, another study showed 47% positive pelvic nodes with positive paraaortic lymph nodes and 31% of patients with positive lymphovascular space invasion (LVSI) and metastasis to paraaortic lymph nodes. In patients with positive paraaortic lymph nodes and LVSI, 0.8% of cases were free of metastasis [6,28]. In our study, we did not have cases with metastases to paraaortic lymph nodes. 

Additionally, the number of removed lymph nodes is important for its diagnostic role. Abu Rustum et al. and Lutman et al. revealed more than 10 lymph nodes in low-risk and high-risk endometrial cancer as diagnostic ones [31,32]. On the other hand, removal of more than 22 pelvic lymph nodes and 10 paraaortic ones was reported by clinicians from Mayo Clinic as diagnostic [33]. In our study, the mean number of paraaortic and pelvic lymph nodes of 17.3 and 24.7, respectively, which stand for diagnostic ones.

What is more, there can be isolated paraaortic nodal recurrence, which is relatively rare in endometrial cancer. However, Abu Rustum et al. described 6% recurrence in paraaortic lymph nodes in patients after previous surgical treatment [34,35]. 

In a review, Salman et al. described sentinel lymph node mapping in high-grade endometrial cancer. Risk of metastases in paraaortic lymph nodes in high-grade endometrial cancer is higher, so it is crucial to carry out staging in this area. The detection rate of sentinel lymph nodes in paraaortic nodes is over 90%. However, for better identification of sentinel lymph nodes of paraaortic localization, uterine injection of indocyanine green is better than intracervical injection (26.8% vs. 6.7%) [36].

Zhang et al. analyzed 1219 cases of endometrial cancer, assessing their characteristics and their prognoses, as these were associated with lymph node metastasis. Their study revealed a higher rate of metastasis in paraaortic and pelvic nodes (56.7%) than pelvic alone (24.2%) or paraaortic alone (19.2%) [37]. There was a proportionally higher rate of metastasis in postmenopausal women than younger women. Positive lymph nodes were correlated with tissue histological differentiation, lymphangitic infiltration, cervical invasion, and deep myometrial invasion and level of CA125. In our study, we did not assess CA125 level, but the involvement of lymph nodes was found to be associated with LVSI and cervical and myometrial invasion.

There are many difficulties in the surgery of paraaortic lymphadenectomy in endometrial cancer. Nakao et al. reported statistically significant differences of operative time and visceral fat area (*p* = 0.026) and periaortic abdominal fat area (*p* = 0.007). They did not observe a correlation between the operative time and body mass index. In our study, we observed a statistically significant association between BMI and pelvic and paraaortic lymph nodes (*p* < 0.05).

In their analysis, periaortic abdominal fat area seemed to be a significant independent marker for the prediction of prolonged operative times for extraperitoneal lymphadenectomy in endometrial cancer. What is more, a high-visceral-fat area is a risk factor of postoperative complications. Otherwise, a periaortic-abdominal-fat area and a limited-visceral-fat area affected operative times without decreasing the number of harvested paraaortic lymph nodes [38]. In our study, we observed an increased number of lymph nodes with higher BMI but no correlation with longer operation time.

On the other hand, Dowdy et al. found that a higher BMI was not correlated with operative time for extraperitoneal laparoscopic paraaortic lymphadenectomy and the most influential factor might be the volume of visceral fat, including the intestinal tract and perivascular fat [39]. 

Completed lymphadenectomy has a crucial role in gynecologic oncology, among other things for staging of the disease. Final histological results depend on further adjuvant treatment. Todo et al. described ultrastaging in paraaortic lymphadenectomy to detect micrometastasis in lymph nodes. Only 15 patients were included in the study; in 4 cases, ultrastaging was negative, 2 had macrometastasis, and in the rest, isolated tumor cells were in paraaortic lymph nodes. Ultrastaging provides more details about lymph nodes and can help choose the best adjuvant treatment. In the study by Todo et al., in five cases, recurrence was observed within 2 years after negative ultrastaging [40]. In our study, no ultrastaging was performed. Nevertheless, complete lymphadenectomy was performed. 

Nowadays, molecular classification is used for selection for endometrial cancer treatment. Jamieson et al. conducted a study with retrospectively assigned molecular classification. There was a correlation of lymph node involvement and p53 mutation in 44.8% of cases, POLE mutation in 14.9% of cases, and NSMP (no specific molecular profile) in 10.8% of cases. There was no involvement of paraaortic lymph nodes [41]. In our study, we planned for respective analysis of gene mutation, because, at the time of performing the study, there was no possibility for an assessment of the molecular classification to be carried out. This will be the most crucial role of lymphadenectomy.

## 5. Conclusions

The robot-assisted approach is a good surgical method for lymphadenectomy for obese patients. The type of docking is not associated with the quality of lymphadenectomy. However, there was a correlation between the number of lymph nodes and the BMI of the patients. 

On the other hand, operation time did not depend on BMI in the robot-assisted mini-invasive approach. Completed pelvic and paraaortic lymphadenectomy allow for staging in high-grade endometrial cancer; however, more investigations are necessary to ascertain the risk of metastases in paraaortic lymph nodes correlated with molecular classification. 

## Figures and Tables

**Table 1:** Characteristics of analyzed population according to FIGO classification.

Staging of Endometrial Cancer	
Mean age (range)	60.07 (34.69–83.23) years
FIGO surgical stage	
1A	12
1B	7
2	4
3A	0
3B	2
3C1	0
C32	0
4	0
Histology	
Endometrioid	21
Serous	1
Clear	2
Mixed	1
Myometrial invasion	
<1/2	12
>1/2	13
Pelvic lymph node metastasis	
Negative	23
Positive	2
Paraaortic lymph node metastasis	
Negative	0
Positive	0

**Table 2:** Comparison of single and dual docking.

Title 1	Single Docking	Dual Docking	Statistical Significance *p* < 0.05
Number of patients	8	17	-
Age [years]	57.48 (34.69–83.24)	61.3 (42.08–73.68)	-
Operation time [min]	173 (110–235)	204 (130–295)	*p* > 0.05
Mean BMI [kg/m^2^]	28.03 (17.97–37.83)	28.59 (19.15–41.52)	*p* > 0.05
Blood loss [mL]	50 (20-100)	49.7 (5–150)	*p* > 0.05
Mean number of paraaortic lymph nodes (range)	15.87 (6–24)	18.05 (7–35)	*p* > 0.05
Mean number of pelvic lymph nodes (range)	24.5 (14–43)	24.88 (11–50)	*p* > 0.05
Abdominal surgery in the past	1	7	*p* > 0.05
Drainage [number of cases]	2	14	-
Complications (range)	0	0	-
Number of involvements of paraaortic lymph nodes	0	0	-
Number of involvements of pelvic lymph nodes	0	2	-

## Data Availability

The data presented in this study are available on request from the corresponding author.
